# The impact of Obstructive Sleep Apnea Hypopnea syndrome severity on depression and anxiety disorders

**DOI:** 10.1192/j.eurpsy.2024.871

**Published:** 2024-08-27

**Authors:** A. Fki, A. Ben Lazreg, R. Bouchech, R. Ben Cheickh, G. Sakly

**Affiliations:** ^1^occupational medicine, Sahloul university hospital; ^2^Faculty of medicine; ^3^department of neurophysiology, Sahloul university hospital, Sousse, Tunisia

## Abstract

**Introduction:**

Obstructive sleep apnea hypopnea syndrome (OSAHS) is a chronic source of stress that can alter the emotional state of affected patients.

**Objectives:**

This study aimed to assess the impact of OSAHS severity on depression and anxiety disorders in a Tunisian population of apneic patients.

**Methods:**

We conducted a cross-sectional study, involving 40 patients diagnosed with OSAHS by polysomnography in the Sleep unit, department of Neurophysilogy at Sahloul university hospital in Sousse, Tunisia. Anxiety and depressive disorders were detected using the Arabic version of the HADS (Hospital Anxiety and Depression Scale).

**Results:**

The mean age was 49.7 ± 7.87 years with a sex ratio of 1.1. The mean apnea-hypopnea index (AHI) was 29.72. OSAHS was mild, moderate and severe in 40%, 22.5% and 37.5% of cases respectively. One third (30%) of patients received a treatment with continuous positive airway pressure (CPAP). The prevalence of depression in the study’s patients, according to the HADS, was 56.4% and that of anxiety was 59%. There was a positive linear relationship between AHI and scores of depression and anxiety (p=0.045 and p=0.037 respectively). Similarly, a significant association was found between HAD scores and treatment with CPAP (p<0.05).

**Conclusions:**

These results show a high frequency of anxiety-depressive disorders in patients with OSAHS. Severity of OSAHS and CPAP treatment proved to be determining factors in anxiety and depressive disorders, hence the importance of detecting these disorders in order to improve patients’ quality of life.

**Disclosure of Interest:**

None Declared

